# Pin1 Inhibitor Juglone Exerts Anti-Oncogenic Effects on LNCaP and DU145 Cells despite the Patterns of Gene Regulation by Pin1 Differing between These Cell Lines

**DOI:** 10.1371/journal.pone.0127467

**Published:** 2015-06-03

**Authors:** Ryuhei Kanaoka, Akifumi Kushiyama, Yasuyuki Seno, Yusuke Nakatsu, Yasuka Matsunaga, Toshiaki Fukushima, Yoshihiro Tsuchiya, Hideyuki Sakoda, Midori Fujishiro, Takeshi Yamamotoya, Hideaki Kamata, Akio Matsubara, Tomoichiro Asano

**Affiliations:** 1 Department of Medical Chemistry, Division of Molecular Medical Science, Graduate School of Biomedical Sciences, Hiroshima University, 1-2-3 Kasumi, Minami-ku, Hiroshima City, Hiroshima Japan; 2 Division of Diabetes and Metabolism, The Institute for Adult Diseases, Asahi Life Foundation, 1-6-1 Marunouchi, Chiyoda-ku, Tokyo, Japan; 3 Department of Internal Medicine, Graduate School of Medicine, University of Tokyo, 7-3-1 Hongo, Bunkyo-ku, Tokyo, Japan; 4 Department of Urology, Graduate School of Biomedical and Health Sciences, Hiroshima University, Hiroshima, Japan; University of Kentucky College of Medicine, UNITED STATES

## Abstract

**Background:**

Prostate cancer initially develops in an androgen-dependent manner but, during its progression, transitions to being androgen-independent in the advanced stage. Pin1, one of the peptidyl-prolyl *cis/trans* isomerases, is reportedly overexpressed in prostate cancers and is considered to contribute to accelerated cell growth, which may be one of the major factors contributing to their androgen-independent growth. Thus, we investigated how Pin1 modulates the gene expressions in both androgen-dependent and androgen-independent prostate cancer cell lines using microarray analysis. In addition, the effects of Juglone, a commercially available Pin1 inhibitor were also examined.

**Methods:**

Two prostate cancer cell-lines, LNCaP (androgen-dependent) and DU145 (androgen-independent), were treated with Pin1 siRNA and its effects on gene expressions were analyzed by microarray. Individual gene regulations induced by Pin1 siRNA or the Pin1 inhibitor Juglone were examined using RT-PCR. In addition, the effects of Juglone on the growth of LNCaP and DU145 transplanted into mice were investigated.

**Results:**

Microarray analysis revealed that transcriptional factors regulated by Pin1 differed markedly between LNCaP and DU145 cells, the only exception being that Nrf was regulated in the same way by Pin1 siRNA in both cell lines. Despite this marked difference in gene regulations, Pin1 siRNA and Juglone exert a strong inhibitory effect on both the LNCaP and the DU145 cell line, suppressing *in vitro* cell proliferation as well as tumor enlargement when transplanted into mice.

**Conclusions:**

Despite Pin1-regulated gene expressions differing between these two prostate cancer cell-lines, LNCaP (androgen-dependent) and DU145 (androgen-independent), Pin1 inhibition suppresses proliferation of both cell-lines. These findings suggest the potential effectiveness of Pin1 inhibitors as therapeutic agents for prostate cancers, regardless of their androgen sensitivity.

## Introduction

Peptidyl-prolyl *cis/trans* isomerase Pin1 is an enzyme that specifically binds to the motifs containing phosphorylated serine or threonine, immediately preceding proline, in numerous proteins. The association with Pin1 promotes cis/trans isomerization of the peptide bond [1–3], and thereby alters their functions [4], stability and/or subcellular localization [5]. Consequently, Pin1 has been shown to be involved in the regulation of many cellular events, including proliferation [6], survival of neurons [7], differentiation [8], metabolism [9–11] and so on. While the expression of Pin1 is ubiquitous, previous reports have shown high levels of Pin1 expression in a number of human malignancies, including lung, breast, colon and prostate cancers [12–15]. Indeed, Pin1 activates numerous oncogenes or growth enhancers and also inactivates a large number of tumor suppressors or growth inhibitors [16]. Thus, ablation of Pin1 reportedly prevents cell growth, or affects various properties including drug sensitivity, motility and metastasis [17].

Prostate cancer is one of the most common male tumors and its incidence has been steadily increasing worldwide [18]. Most prostate cancers have the characteristics of androgen-dependent cell growth [19] and androgen-deprivation therapy in advanced prostate cancer is currently used in clinical practice. However the majority of patients eventually develop resistance and progress to castration-resistant prostate cancer (CRPC) [20,21]. Therefore, it is likely that gene alterations leading to androgen independence and cellular growth gradually accumulate during the progression of prostate cancers [22].

On the other hand, Pin1 reportedly plays an important role not only in tumorigenesis but also in maintenance of the transformed phenotype in prostate cancer cells [23]. However, genes of which the expressions are regulated by Pin1 have not yet been identified in prostate cancers. In this study, we used two prostate cancer cell line types, LNCaP which has an androgen dependent growth property, and DU145 which shows androgen independent growth, and compared the genes regulated by Pin1 between these two cell lines.

In addition, we investigated the effects of Juglone, an inhibitor of Pin1, on the proliferations of LNCaP and DU145 cells *in vitro* as well as when inoculated into mice. Juglone is an inhibitor of Pin1 isolated from walnut skin, by screening a collection of pure secondary metabolites against the PPIase activity of E. coli parvulin [24]. In some human malignancies including breast cancer, leukemia and gastric cancer, Juglone has been reported to inhibit cell growth [25–28]. However, it should be noted that Juglone is likely to inhibit molecules other than Pin1, as Juglone reportedly causes tubulin aggregation or the disappearance of BubR1 immunoreactivity [29]. Thus, there are undoubtedly differences between Pin1 siRNA and Juglone treatments. We herein show Pin1-regulated gene expressions to differ between these cell lines, though Juglone still exerts an anti-oncogenic effect on both, which raises the possibility of Pin1 as a therapeutic target in prostate cancers.

## Materials and Methods

### Cell Lines and Culture Conditions

The prostate cancer cell lines LNCaP and DU145, purchased from American Type Culture Collection (Manassas, VA), were maintained in RPMI 1640 (Nissui Pharmaceutical, Tokyo, Japan) and DMEM, respectively, containing 10% (vol/vol) fetal calf serum at 37°C in 5% CO_2_ in air. The Pin1 inhibitor Juglone was purchased from EMD Chemicals Inc. (San Diego, CA). All other reagents were of analytical grade.

### Small interfering RNA transfection

For the knockdown of human Pin1, the siRNAs against Pin1 (Pin1 shRNA-1: 5’-CGGCAACAGCAGCAGUGGUGGCAAA-3’ and Pin1 shRNA-2: 5’-GCCCUGGAGCUGAUCAACGGCUACA-3’) and control siRNA were purchased from Invitrogen (Stealth/siRNA duplex oligoribonucleotides), and transfected into LNCaP or DU145 cells using Lipofectamine RNAi Max (Invitrogen, CA), according to the manufacturer’s instructions.

### Western Blot Analysis

The anti-ß-actin (1:2000) antibody was purchased from Sigma (St. Louis, MO), the anti-Pin1 (1:1000) antibody from Santa Cruz Biotechnology (Santa Cruz, CA), and the anti-rabbit/mouse horseradish peroxidase (HRP) secondary antibody (1:2000) from GE Healthcare (Little Chalfont, UK). The cells were solubilized with Laemmli buffer (0.2 M Tris·HCl, 4% SDS, 10% glycerol, 5% 2-mercaptoethanol and 0.1% bromophenol blue). Equal amounts of protein from whole cell lysates were resolved by SDS-PAGE. The proteins were then transferred to polyvinylidene difluoride membranes (Millipore, MA). The membranes were blocked with 3% nonfat dry milk or 5% bovine serum albumin in Tris-buffered saline with 0.1% Tween 20 and incubated with specific antibodies, followed by incubation with HRP-conjugated secondary antibodies. The antigen-antibody interactions were visualized by incubation with ECL chemiluminescence reagent (GE Healthcare).

### Microarray analysis

At 48 h after the transfection of Pin1 or control siRNAs, total RNA was isolated from LNCap and DU145 cells using Trizol, followed by the RNAeasy kit (Quiagen, Crawley, UK). The quantity and quality of the extracted RNA were checked with a 2100 Bioanalyzer (Agilent Technologies, Santa Clara, CA) using an Agilent RNA 6000 Nanokit. Cells treated with the control were compared to those with knockdown of Pin1.

Human gene 1.0 ST arrays were used to identify genes expressed differently in LNCap and DU145 in response to Pin1 knockdown. The arrays were scanned using the Affymetrix GeneChip Scanner 3000 7G controlled by GeneChip Operating Software, 1.3 (Affymetrix, CA). The significant gene expression changes were extracted by deleting genes with low expression levels or low reliability. The resultant gene expressions were normalized employing the RMA16 algorithm in GeneSpring 12 (Agilent Technology). Gene lists were formatted and uploaded to Ingenuity^R^ Pathway Analysis (IPA, TOMY digital biology, Inc., Tokyo, Japan), and genes with gene ontology (GO) such as positive and negative cell proliferation, angiogenesis, cell cycling and apoptosis were identified. Genes of which expression levels were altered by both Pin1 shRNA-1 and -2, i.e. showed a more than 1.5-fold change (FC), were identified. Probe sequences and other details on the microarray platform can be found in the GEO database (http://www.ncbi.nlm.nih.gov/geo/) under accession number GSE67457.

### Analysis of gene expression changes

The transcription factors regulating genes which were changed by Pin1 knockdown were analyzed using KeyMolnet Lite ver. 5.4 (KM data Co., Japan). The gene lists normalized by GeneSpring 12 were imported to the software, and scores(p) were calculated employing the following equations, from probability based on a hypergeometric distribution [30]

$$score(p)\;=\;\sum_{x\;=\;0}^{Min(C,V)}f(x)$$

$$f\left(x\right)\;=\;CCx*T-CCV-x/TCV$$

O, T, C and V were as follows:

O was the number of overlaps between molecules regulated by the transcriptional factor and resultant items, indicating altered expression, i.e. exceeding 1.5-FC. T was the total number of molecules in KeyMolnet Lite. C was the number of molecules regulated by the transcriptional factor. V was the number of resultant items. Resultant transcriptional factors were listed by ascending order based on their scores(p), and the top 8 transcriptional systems were taken to be arbitrary.

### Quantitative PCR

RNA extraction was performed using Sepasol (Nakalai Tesque, Tokyo, Japan) according to the manufacturer's protocol. Briefly, cells were homogenized in Sepasol, and chloroform was added. After being vortexed, the tubes were centrifuged at 15,000 rpm for 10 min. Upper phases were transferred to new tubes, and total RNA was obtained by ethanol precipitation. cDNA was obtained using total RNA employing a Verso cDNA synthesis kit (Thermo Scientific, Yokohama, Japan). Reaction solutions contained 5× cDNA synthesis buffer, dNTP mix, oligo(dT) primer, RT enhancer, Verso enzyme mix, and 1 μg of total RNA. The program for the reverse transcription cycle was 42°C for 30 min and 95°C for 2 min. Real time quantitative PCR was carried out using a CFX96 real time PCR system (Bio-Rad, CA) with SYBR mix (Takara Bio, Inc, Shiga, Japan), according to the manufacturer's protocol. Reaction solutions were 20 μl and contained SYBR premix EX taq, primers, and template. PCR was carried out in two steps, at 95°C for 5 s and 60°C for 30 s, repeated 40 times. Relative mRNA genes were normalized to the GAPDH mRNA level and relative expression levels determined by the comparative Ct method.

### MTT assay for the cells treated with Pin1 siRNA or Juglone

LNCaP or DU145 cells were seeded in a 96-well plate (100 μl/well) for 24 h. Then, the cells were treated with Pin1 siRNA or various concentrations of Juglone (5, 10, 15 and 20 μM) for 48 h. The viability of the cells treated with Pin1 siRNA or Juglone was evaluated by MTT assay using MTT (3-[4,5-dimethylthiazol-2-yl]-2,5- diphenyltetrazolium bromide; thiazolyl blue). Briefly, 0.5mg/ml MTT was added to each well for 3 h at 37°C in 5% CO2. After the medium had been removed to a 96-well plate (100 μl/well), absorbance at 490 nm was recorded using a GloMax-Multi Microplate Multimode Reader (Promega, WI).

### Mouse xenograft model

The animal protocol was approved by the Institutional Animal Care Committee of Hiroshima University. Male athymic nu/nu mice (6 weeks old, NCI-Frederick) were housed under climate-controlled conditions with a 12:12-h light/dark cycle and were provided standard food and water *ad libitum*. All protocols were approved by the Institutional Review Board of Hiroshima University.

The nu/nu male mice were used for tumor inoculation. Briefly, prostate cancer cells of the DU145 line were inoculated subcutaneously into the backs of the mice (2x10^6^ cells in 200 μl PBS/site). Three weeks after tumor inoculation, the mice were divided into 2 groups (8 mice per group). Those in the treatment group were intraperitoneally injected with Juglone once a week for 4 weeks (40 μg/mouse), while mice in the control group were injected with 0.9% sodium chloride. Tumor size was determined twice a week by measuring tumor length and width with calipers and volumes were determined using the formula volume = length x width ^2^ /2. The mice were also weighed twice a week.

### Statistical Analysis

The data are expressed as means ± standard deviation (SD). The differences between the two groups were assessed using unpaired Student’s t-tests. Values of p < 0.05 were considered to indicate statistically significant differences. Analyses were carried out using JMP software (version 10.0; SAS Institute, Cary, NC, USA) and Microsoft Excel 2013.

## Results

### Gene expressions regulated by Pin1 in LNCap and DU145 cells

**Fig 1:**
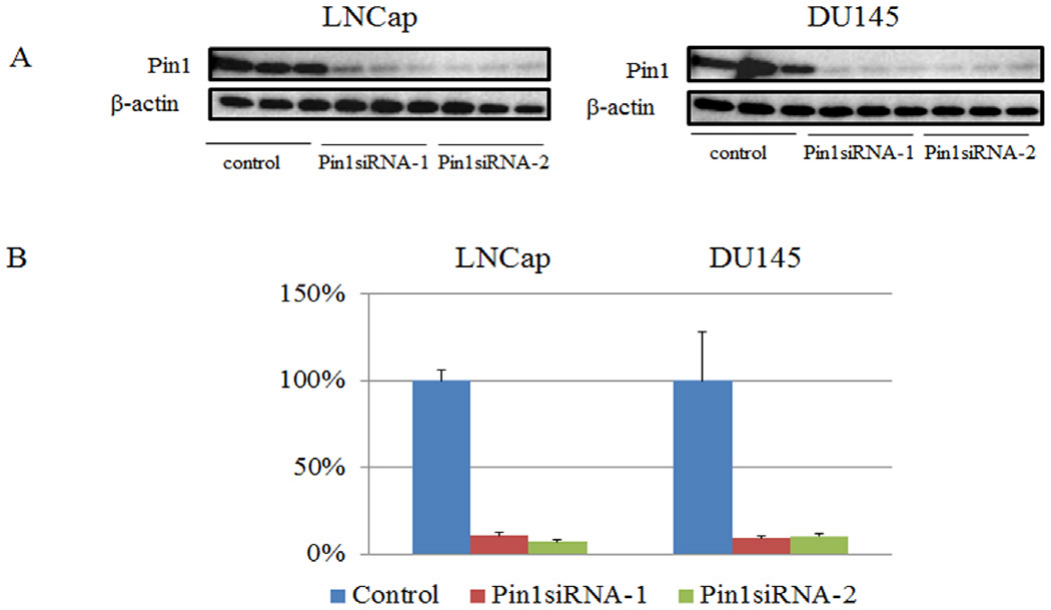
A. Western blotting using anti-Pin1 antibody and anti- ß-actin antibody as internal controls. LNCap and DU145 cells are transduced with control-siRNA or Pin1 siRNA for 48hr. B. Quantification of bands in Fig 1A. Bars indicate means±S.E. for the ratio of the band intensity of Pin1 to that of ß-actin.

Pin1 protein expressions in LNCap and DU145 cells were reduced by approximately 90% by treatment with either Pin1 siRNA-1 or siRNA-2 (Fig 1). The mRNAs prepared from LNCap and DU145 treated with either one of the two Pin1 siRNAs or control siRNA were subjected to microarray analysis. The genes with more than 1.5-FC in their expressions induced by Pin1 siRNAs were identified. There were 3092 genes showing increased expression and 2369 showing decreased expression, in total in the two cell lines. IPA software was used to categorize the cancer related genes of which expressions were significantly changed (≧1.5-FC) by Pin1 knockdown in the IPA database. Table 1 summarizes the results for the genes belonging to the GO categories of cell proliferation, angiogenesis, cell cycling and apoptosis. The genes of which expression levels were altered in both LNCap and DU145 were revealed to be GRB2, HEATR1, IFT57, and SGMS2.

**Table 1:** The genes belonging to the GO categories of cell proliferation, angiogenesis, cell cycling and apoptosis; FC: fold change.

	LNCap	DU145	LNCap & DU145
Positive regulation of cell proliferation FC<0.67	TOMM40L|NR1I3,CAMK2N1,KIF20B,ACSL5,EFNB2,FNTB,MT1E,GIT1,KLK3,SIRT2,KLK4,IL1RN,PAX1,FERMT1,HMGA1,HIP1,EXTL3	TACSTD2,PγGO2,CLCF1,RPS6KA5,CLCF1,ADA,CSNL1G3,IGF2BP3	0
Negative regulation of cell proliferation FC>1.5	DNAJB4,CSF1,PTPN14,ESRRG,TNKS2,BLNK,FGFR2,ITGA5,FLT3,PPM1A,SPRED1,NDN,DCUN1D3|LYRM1,GRB2,TCF4,GRB14,IGFBP5,RHOH,HPGD,F2RL1,SESN1,PARK2,AHR,GLI3,CDK6,CD274,DAPK1,TGFBR1,RLN2,HLA-DRB3	GRB2,MAP3K1	GRB2
Positive regulation of angiogenesis FC<0.67	EFNB2,TM4SF1	0	0
Negative regulation of angiogenesis FC>1.5	FGFR2,AHR,TGFBR1	0	0
Positive regulation of cell cycling FC<0.67	KIF20B,FNTB,SIRT2,TM4SF1,HMGA1	0	0
Negative regulation of cell cycling FC>1.5	DNAJB4,PBX1,FGFR2,PTPRB,DCUN1D3|LYRM1,IGFBP5,PLAC8,HPGD,F2RL1,AHR,CDK6,CD274,TGFBR1,CDC14B	0	0
Negative regulation of apoptosis FC<0.67	TOMM40L|NR1I3,EFNB2,MT1E,KLK3,SIRT2,IL1RN,RND3,GALNT3,PAX1,MME,HMGA1,HIP1	CLCF1	0
Positive regulation of apoptosis FC>1.5	S100PBP|YARS,SH3GLB1,CSF1,PPP2R5A,MCOLN3,HEATR1|LGALS8,TNKS2,BLNK,FGFR2,GAS2,DRAM1,ITGA5,FLT3,PPM1A,SERPINA3,DCUN1D3|LYRM1,GRB2,TCF4,GCG,IGFBP5,FAM162A,RHOH,ALB,SGMS2,ANTXR2,PLAC8,SLC7A11,F2RL1,STK38,PARK2,AHR,GLI3,CDK6,SDC2,CD274,DAPK1,TGFBR1	HEATR1,GRB2,IFT57,SGMS2,MAP3K1	HEATR1,GRB2, IFT57,SGMS2

**Fig 2:**
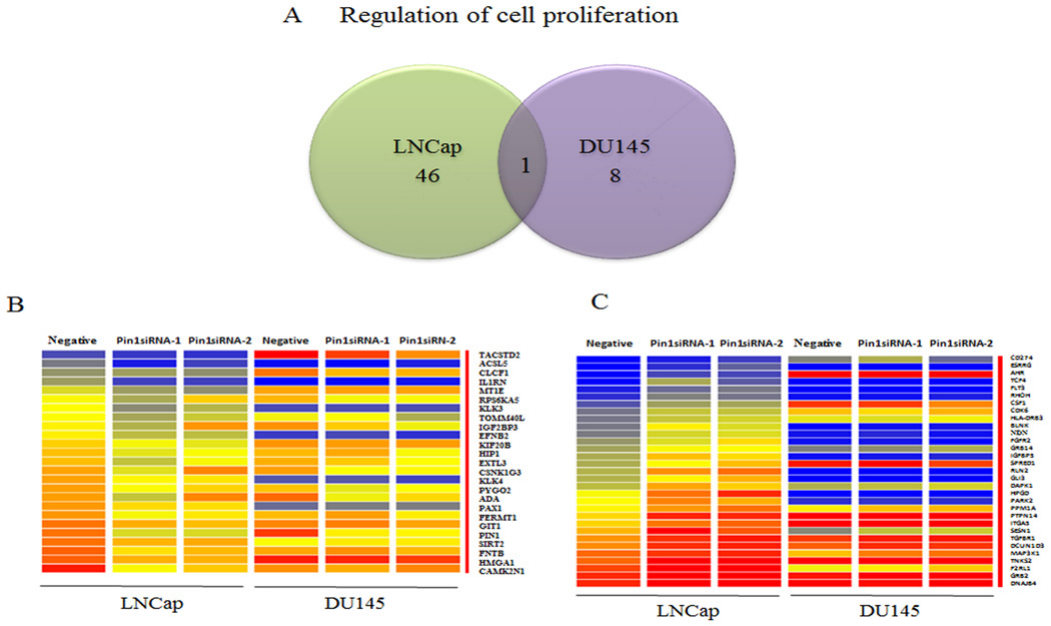
A. Venn diagram showing the number of genes positively or negatively regulated, as reflected by cell proliferation being down- or up-regulated (1.5<FC), respectively, by Pin1 knock-down in LNCap and DU145 cells. B and C. Heat map presentation of genes expressed differently in LNCap and DU145 cells. In terms of cell proliferation, 25 positively regulated and 31 negatively regulated genes show the same expressions in response to the two different siRNAs.

Fig 2 summarizes the results focusing on the genes belonging to the category of cell proliferation, among those with increased and decreased expressions. While the number of genes changed in DU145 by Pin1 siRNAs is obviously less than that in LNCap, there is little overlap in these genes (Fig 2A). A heat map, indicating genes expressed differently in LNCap and DU145 cells, revealed approximately 25 genes to be upregulated (Fig 2B) and 31 to be downregulated (Fig 2C), during cell proliferation.

**Fig 3:**
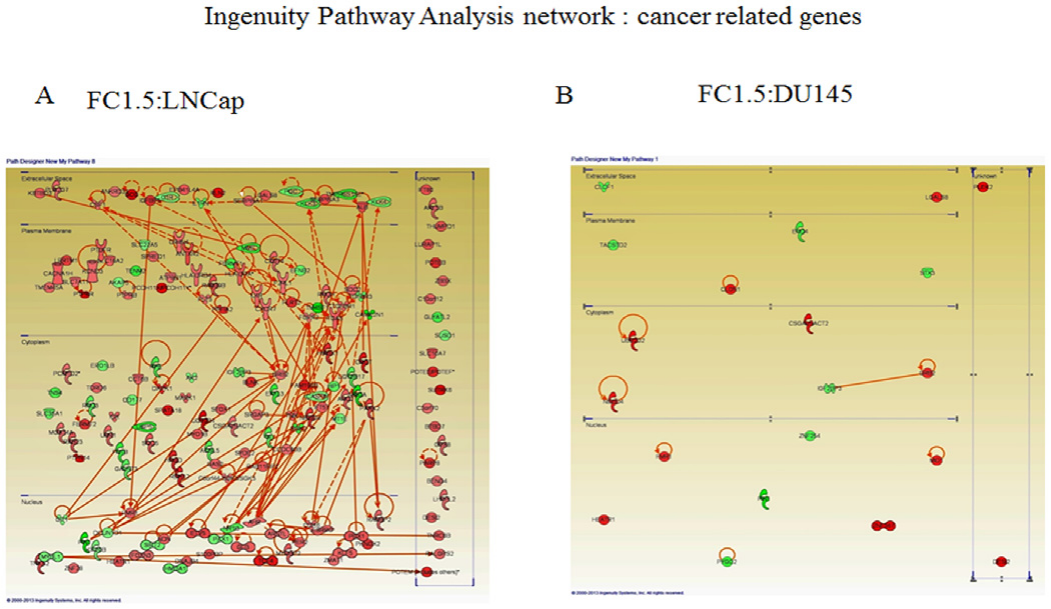
Ingenuity Pathway Analysis network depicting relationships among cancer related genes expressed differently in LNCap (3A) or DU145 (3B) cells. Intensity of the red color indicates the degree of upregulation. Intensity of the green color indicates the degree of downregulation. Nodes are displayed using various shapes that represent the functional class of the gene product. Edges are displayed with various labels that describe the nature of the relationship between the nodes: a continuous line indicates a direct relationship; broken lines represent indirect relationships; an arrow indicates that one gene product acts on another.

The expression levels of many cancer related genes localized in the extracellular space, plasma membrane, cytoplasm or nucleus were shown to be significantly changed in LNCap cells, while fewer such genes were detected in DU145 (Fig 3A), when mapped employing IPA software. For example, KLK3 encoding prostate-specific antigen (PSA), TM4SF1 known to be a tumor-associated antigen, FGFR2 known to be related to growth and tumorigenicity, IFT57 among the apoptosis related genes, HIP1 as a cellular survival factor in prostate cancer, and the like, were altered by Pin1 knockdown. Furthermore, IPA revealed numerous genes, including these prostate cancer related genes, to form a complex network in LNCap cells. On the other hand, fewer genes involved in cancer related pathways were detected in DU145 cells.

### Difference in transcriptional regulation by Pin1 between LNCap and DU145 cells

KeyMolnet Lite was employed for the analysis of transcription factors, activities of which were estimated to be altered by inhibition of Pin1 expression. Table 2 shows the top 8 transcription factors located downstream from Pin1 in LNCap and DU145, respectively. The transcription factors with scores(p) < 0.05 belonged to the Ets-domain family and E2A/EBF/PAX2 in LNCap, and were Nrf, MRF, TR, CPEB, KLF, C/EBP, MEF and HIF in DU145 (Table 2). The score(p) was higher for LNCap than DU145, according to the KeyMolnet algorithm, because more genes were changed in LNCap than in DU145.

**Table 2:** Analysis of transcription regulation using KeyMolnet Lite; The top 8 transcription factors are located downstream from Pin1 in LNCap.

Transcriptional factor	p-value
Ets-domain family	5.48E-03
E2A/EBF/PAX2	3.69E-02
Myc	6.07E-02
FXR	6.12E-02
DBP	6.63E-02
Nrf	6.74E-02
p53	8.11E-02
Androgen Receptor	1.06E-01

**Fig 4:**
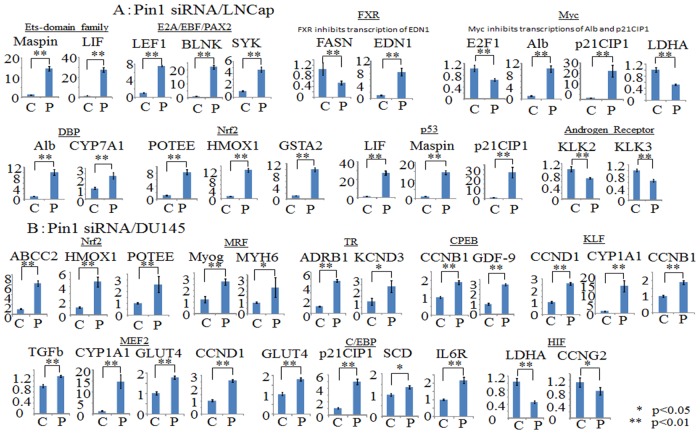
Real-time PCR is applied and relative gene expression levels are determined by the comparative Ct method. Bars indicate the means±S.E. of ratios of the relative expressions of the target mRNA to that of GAPDH. The mean of the control is arbitrarily set at 1. Statistical significance is indicated by * (p<0.05) or ** (p<0.01). A. LNCap cells were transduced with control-siRNA (C) or Pin1 siRNA (P) for 48hr. B. DU145 cells were transduced with control-siRNA (C) or Pin1 siRNA (P) for 48hr.

Subsequently, we investigated regulations of the genes downstream from the transcription factors identified in Table 2, using the RT-PCR method. As shown in Fig 4A, in LNCap cells, the downstream genes of the Ets-domain family, E2A/EBF/PAX2, DBP, Nrf and p53 were significantly upregulated by Pin1 shRNA-1 knockdown, while those of Myc, FXR and the Androgen Receptor were downregulated. On the other hand, in DU145 cells, as shown in Fig 4B, the downstream genes, i.e. Nrf, MRF, TR, CPEB, C/EBP and MEF2, were upregulated by Pin1 knockdown, while KLF and HIF were downregulated.

**Fig 5:**
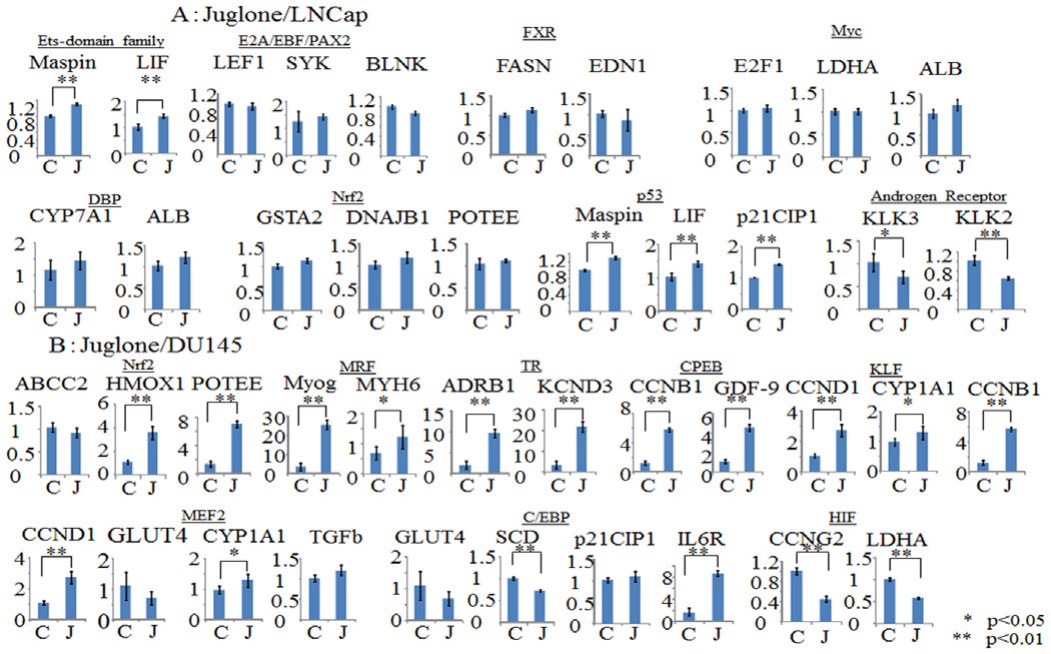
Real-time PCR is applied and relative gene expression levels are determined by the comparative Ct method. Bars indicate the means±S.E. of the ratios of the relative expressions of the target mRNAs to that of GAPDH. The mean of the control is arbitrarily set at 1. Statistical significance is indicated by * (p<0.05) or ** (p<0.01). A. LNCap cells were incubated with control solvent (C) or 10 μM Juglone (J) for 48hr. B. DU145 cells were incubated with control solvent (C) or 10 μM Juglone (J) for 48hr.

Furthermore, we investigated whether the gene expressions altered by the Pin1 inhibitor Juglone were similar to those induced by Pin1 siRNA. Fig 5A and 5B show the gene expression alterations when cells were incubated with 10μM Juglone for 48 hours, for the same genes as presented in Fig 4. Although there were some differences, the alterations in the expression levels of the genes downstream from the Ets-domain family, DBP, Nrf, p53 and the androgen receptor were similar with Pin1 siRNA and Juglone treatments, in the LNCap cell line (Fig 5A). In DU145 cells, the regulations of the MRF, TR, CPEB, KLF and HIF genes were shown to be similar with Pin1 siRNA and Juglone treatments (Fig 5B).

### Knockdown of Pin1 by siRNA or treatment with Juglone inhibits the growth of LNCap and DU145 cells, *in vitro* and *in vivo*

**Fig 6:**
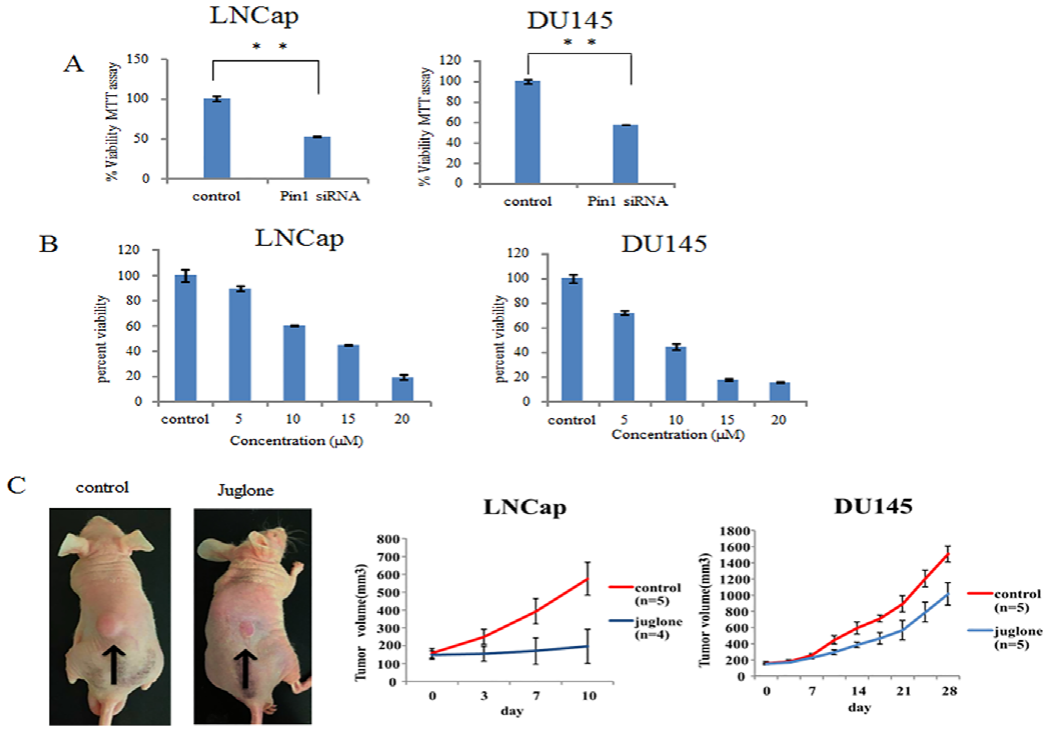
Pin1 inhibition suppresses cell growth in LNCap and DU145 cells. A. LNCap and DU145 cells were transduced with control-siRNA or Pin1 siRNA for 48hr. The *in vitro* cell proliferation inhibitory potential of Pin1 knockdown in the two cell lines was assessed employing the MTT assay. B. The *in vitro* cell proliferation inhibitory potential of 10 μM Juglone administration for 48 hour in the two cell lines was assessed employing the MTT assay. C. Effect of Juglone on tumor growth in mice inoculated with LNCap cells. Three weeks after tumor inoculation, the mice were divided into 2 groups (8 mice per group). Those in the treatment group were intraperitoneally injected with Juglone once a week for 4 weeks (40μg/mice), while mice in the control group were injected with solvent (0.9% NaCl).

Treatment with Pin1 siRNA significantly inhibited cell proliferation, as judged by the MTT assay, in both LNCap and DU145 cells (Fig 6A). Similarly, the viability of LNCap and DU145 cells was suppressed by Juglone treatment (Fig 6B), and the Juglone effect was concentration-dependent. LNCap was slightly resistant to Juglone, at a lower concentration, as compared with DU145.

Furthermore, to investigate the effect of Juglone on cell proliferation in vivo, we examined its effects in a mouse xenograft model. As shown in Fig 6C, the tumors derived by LNCap cell inoculation in the control group showed rapid and massive growth while intraperitoneal Juglone administration essentially inhibited tumor growth from the beginning through day 10. The tumors comprised of DU145 cells in the control group showed slow increases within the initial 7-day observation period, and after day 11, a rapid acceleration was observed in the control group, with the size increasing from 157 mm^3^ on day 1 to reach 1508 cm^3^ in 28 days (9.6-fold increase). The Juglone treatment did not affect tumor sizes during the first 7 days, but after day 7 and up to day 28, cell growth was significantly suppressed as compared with the control. The body weights of mice were not changed by Juglone treatment.

## Discussion

Over the past few decades, a considerable number of studies have established the important role of Pin1 in oncogenesis [31–34]. Pin1 inhibition reportedly blocks the cell growth of prostate cancer cell lines [35], and Pin1 expression in prostate cancer is regarded as an independent prognostic marker [14]. Furthermore, Pin1 depletion in athymic mice inhibits both tumor growth and angiogenesis [23].

We identified the genes of which expressions are regulated by Pin1, and classified them into the four categories (cell proliferation, angiogenesis, cell cycling and apoptosis) according to the IPA database (Table 1). More genes were regulated by Pin1 in LNCap than in DU145 and the patterns of gene regulation differed markedly between these two cell lines (Fig 2). In the LNCap cells, gene expressions are related to growth and/or changes in cellular phenotype including androgen dependency [36–42], as exemplified by KLK3, HMGA1, FGFR2, PPM1A, HPGD, TM4SF1 and SH3GLB1. For example, loss of FGFR2 is reportedly involved in the molecular mechanism underlying androgen insensitivity [43]. One possibility is that Pin1 inhibition may block switching to an androgen independent phenotype via FGFR2 maintenance. The commonly observed genes, such as GRB2, HEATR1, IFT57 and SGMS2, are known to be related to apoptosis [44–47]. Downregulation of TACSTD2 reportedly stimulates tumor growth [48].

The KeyMolnet database and probability statistics, based on a hypergeometric distribution, were used to profile our data from the viewpoint of transcriptional factors (Tables 2 and 3). From the database, it is apparent that most of the genes presented in Fig 4 are upregulated via their regulating transcriptional factors. The exceptions are FXR, which inhibits the transcription of EDN1, Myc which inhibits the transcription of Alb and p21CIP1, and KLF which inhibits the transcription of CCND1, CYP1A1 and CCNB1. Thus, Nrf is the only transcriptional factor regulated by Pin1 in both LNCap and DU145. Nrf2 mediates detoxification of reactive oxygen species [49] and is involved in sensitivity to chemical carcinogenesis induced in FGFR2b knockout mice. Moreover, p53 and the Androgen Receptor in LNCap, as well as HIF in DU145, might be involved in cell growth. For example, Pin1 is known to be a critical regulator of the tumor suppressor p53 during the DNA damage response [23]. The role of the androgen receptor is pivotal, and androgen receptor serine 81 mediates Pin1 interaction and defines tumor grade by modulating androgen receptor function [50]. Juglone reportedly induces apoptosis in LNCap via down-regulation of androgen receptor expression [51].

**Table 3:** Analysis of transcription regulation using KeyMolnet Lite; The top 8 transcription factors are located downstream from Pin1 in DU145.

Transcriptional factor	p-value
Nrf	2.00E-05
MRF	1.34E-03
TR	1.34E-03
CPEB	1.97E-03
KLF	2.76E-03
C/EBP	9.22E-03
MEF2	1.25E-02
HIF	1.38E-02

Finally, we confirmed the cytostatic activity that occurs with Pin1 inhibition in LNCap and DU145 both *in vivo* and *in vitro* (Fig 6). The *in vitro* effects of Pin1 siRNA and Juglone on the MTT assay results were similar. The effects of Juglone in androgen-independent prostate cancer cells have not as yet been studied and proof of its effects is thus lacking. In a xenograft model, LNCap inoculated cells grew rapidly and Juglone almost completely inhibited tumor growth. DU145 cells grew slowly at first, with a gradual acceleration of growth from day 11 onward, and Juglone affected only the latter, more rapid, phase of this growth. We can speculate that the difference between the periods during which Juglone exerted its effects may involve Pin1 regulating systems being critical for more rapid proliferation of tumors. However, it is noteworthy that inoculated DU145 cells still do not grow in Pin1 regulated systems when Juglone administration begins.

In general, there were considerable differences in gene regulation between LNCap and DU145. Although the mechanism of transition to androgen-independent prostate cancer has yet to be clarified, several molecular mechanisms underlie the growth of androgen-independent prostate cancer. For example, the activations leading to the hypersensitive pathway (Androgen Receptor amplification, increased Androgen Receptor sensitivity), the promiscuous pathway (Androgen Receptor mutations, Co-regulator alterations), the outlaw pathway (Growth-factor-activated outlaw pathways, receptor-tyrosine-kinase-activated outlaw pathways, the AKT pathway), the bypass pathway, the lurker cell pathway and so on are all possible contributors to the mechanisms of transition to androgen independence [50]. It can reasonably be speculated that these pathways are involved in the changes in phosphorylation states of several proteins which are targets of Pin1. Moreover representative differences between LNCap and DU145 reportedly include the presence or absence of Androgen Receptor, PTEN, or mutant of p53 [50–52]. Expression of the Androgen Receptor especially is reportedly down-regulated by Pin1 siRNA or Juglone in LNCap [53,54], but DU145 is an Androgen Receptor negative cell-line [55–57]. In addition, the expression of AR genes directly regulates c-Myc transcription [58] and functions as a coactivator of the Ets domain family [59]. These differences in regulations are assumed to be responsible for the differing microarray results, but further studies are necessary to resolve this issue.

We focused mainly on comparative studies employing microarray analysis. One of the major limitations of our study is the possibility that an unknown system beyond the scope of our database is involved in Pin1 regulated oncogenesis. Another limitation is that expressions were based only on mRNA levels as determined by microarray. These results need to be examined in greater detail in the future. However, our system, by detecting statistically significant transcriptional changes, is potentially useful for comprehensively understanding the contributions of specific genes and pathways involved in oncogenesis and cancer treatment.

## Conclusion

Numerous variations in mechanisms and factors, such as androgen receptor mutations, are involved in the transition from an androgen-dependent to an independent state. Despite Pin1-regulated gene expressions differing between these two prostate cancer cell-lines, LNCaP (androgen-dependent) and DU145 (androgen-independent), Pin1 inhibition suppresses the proliferation of both. These findings suggest the potential effectiveness of Pin1 inhibitors as therapeutic agents for prostate cancers, regardless of their androgen sensitivity.
